# Factors Affecting the Performance of HRP2-Based Malaria Rapid Diagnostic Tests

**DOI:** 10.3390/tropicalmed7100265

**Published:** 2022-09-25

**Authors:** Xavier Martiáñez-Vendrell, Malia Skjefte, Ruhi Sikka, Himanshu Gupta

**Affiliations:** 1 Molecular Virology Laboratory, Department of Medical Microbiology, LUMC Center for Infectious Diseases (LU-CID), Leiden University Medical Center, 2333 ZA Leiden, The Netherlands or; 2 Department of Global Health and Population, Harvard TH Chan School of Public Health, Boston, MA 02115, USA; 3 Department of Biotechnology, Institute of Applied Sciences & Humanities, GLA University, Mathura 281406, UP, India

**Keywords:** malaria, RDTs, HRP2, *Plasmodium falciparum*, gene deletions, malaria diagnosis

## Abstract

The recent COVID-19 pandemic has profoundly impacted global malaria elimination programs, resulting in a sharp increase in malaria morbidity and mortality. To reduce this impact, unmet needs in malaria diagnostics must be addressed while resuming malaria elimination activities. Rapid diagnostic tests (RDTs), the unsung hero in malaria diagnosis, work to eliminate the prevalence of *Plasmodium falciparum* malaria through their efficient, cost-effective, and user-friendly qualities in detecting the antigen HRP2 (histidine-rich protein 2), among other proteins. However, the testing mechanism and management of malaria with RDTs presents a variety of limitations. This paper discusses the numerous factors (including parasitic, host, and environmental) that limit the performance of RDTs. Additionally, the paper explores outside factors that can hinder RDT performance. By understanding these factors that affect the performance of HRP2-based RDTs in the field, researchers can work toward creating and implementing more effective and accurate HRP2-based diagnostic tools. Further research is required to understand the extent of these factors, as the rapidly changing interplay between parasite and host directly hinders the effectiveness of the tool.

## 1. Introduction

In 2000, malaria was pinpointed as a major burden for global health and development, and efforts have since increased to reduce malaria cases and associated mortality [1,2]. According to the World Health Organization’s (WHO) World Malaria Report, an estimated 241 million malaria cases occurred worldwide in 2020. Between 2000 and 2020, there was a global reduction in malaria mortality, as deaths decreased from 896,000 in 2000 to 627,000 in 2020 [3]. Such a decrease in mortality goes hand in hand with an upward surge in malaria diagnostic tools—facilitated by the 2010 WHO recommendations on confirming all suspected cases by rapid diagnostic tests (RDTs), microscopy and polymerase chain reaction (PCR) [4]. Since their introduction in the late 1990s, RDTs have considerably facilitated the diagnosis of malaria cases, particularly in regions where good-quality microscopy is not always available, and now represent the primary tool for malaria diagnosis worldwide [5]. During 2010–2020, manufacturers sold approximately 3.1 billion RDTs for malaria diagnosis and almost 81% of these RDTs were used in sub-Saharan African countries, where the largest number of malaria cases and deaths take place [3].

RDTs are field-deployable lateral-flow immunochromatographic tests that can detect *Plasmodium* specific antigens in a drop of fresh blood positive for malaria. The target antigens in current commercially available malaria RDTs are histidine-rich protein 2 (HRP2), specific for *Plasmodium falciparum* (*Pf*), and the parasite enzymes lactate dehydrogenase (pLDH) and aldolase, present in all *Plasmodium* species infective to humans [5]. *Pf* HRP2 is encoded by the *pfhrp2* gene (PlasmoDB gene ID: PF3D7_0831800), which is located subtelomerically on chromosome 8 [6,7,8]. *Pfhrp2* bears significant polymorphism rates [9] and was reported to have an 85–90% homology in nucleotide sequence-flanking repeats with the *pfhrp3* gene [8], encoding a protein (HRP3) thought to cross-react with monoclonal antibodies (mAbs) in RDTs [6]. However, viable isolates lacking the *pfhrp2* gene have been collected globally in recent years, rendering concerns over its implications in malaria diagnostics and case management (previously reviewed in [10,11]). Whether the gene is intact or not, it is translated into a water-soluble glycoprotein and released into the peripheral circulation during schizogony [12,13]. Once released, HRP2 persists for several weeks in the bloodstream, even when the infection is cleared, leading to false-positive RDT results [14,15].

RDTs that detect *Pf*-specific HRP2 are the most commonly used due to their efficient qualities including high thermal stability, sensitivity, specificity, cost effectiveness and easy-to-use abilities [16,17]. The contribution of both HRP2-based RDTs and general RDTs in malaria diagnostics and surveillance is imperative to the detection and future eradication of malaria cases caused by *Pf* and other *Plasmodium* species. Regardless of their noted success, RDTs are not a perfect malaria diagnosis tool as poor product design or quality, inadequate storage, and incorrect use can limit their performance and accuracy [16,18]. The COVID-19 pandemic has added additional stress to malaria diagnosis and its management, with supply chain disruptions affecting RDT availability [19]. As COVID-19 and malaria patients present similar symptoms such as fever, it is recommended that health workers administer RDTs to test for both diseases, although this dual testing may be limited in resource poor settings. Additionally, negative test results for both diseases, either due to resource restraints or asymptomatic cases, may result in continued community transmission [19]. Multiple anecdotal reports have also highlighted cases of false-positive SARS-CoV-2 RDT results among confirmed malaria cases [20], further complicating case management of both diseases in endemic countries.

Overall, the performance of HRP2-targeting RDTs and their usefulness in different transmission settings depends, among other factors, on the interplay between the parasite, its host and the RDT itself. This review seeks to present the factors that engage in this interplay and have an overall influence on RDTs as a tool for malaria diagnosis and case management.

## 2. Parasitic Factors

### 2.1. HRP2 Persistence

Once HRP2 is released into the peripheral circulation during schizogony, it can persist for several weeks in the bloodstream, even when the infection is cleared, leading to false-positive RDT results [12,13,14,15]. Studies suggest that RDTs targeting HRP2 have low specificity for diagnosis of *Pf* malaria as significant yields of false-positive results have been observed, particularly in areas of high transmission [14,21,22]. Such results are attributed to the persistence of the protein in blood circulation even after the parasites have been cleared, which can lead to overmedication of patients and subsequent problems, particularly the emergence of drug-resistant or -tolerant parasite clones.

The first study (1993) to describe HRP2 persistence found detectable antigen levels in the blood of 10 cases (25%) 5 days after treatment in a Tanzanian longitudinal cohort, 4 of which remained positive for 14 days [23]. Later studies detected circulating HRP2 for divergent time periods, ranging from a few days up to several weeks [14,22,24,25]. However, the kinetics of HRP2 antigenemia are not well understood, although important indicators, such as production rates and elimination half-life, have been estimated in both in vitro and in vivo studies [26,27]. A model of HRP2 kinetics was applied to clinical data from two studies on human infection, which indicated that in malaria naïve hosts, *Pf* parasites of the 3D7 strain produce 1.4 × 10^−13^ g of HRP2 per parasite for each replication cycle. In addition, it was demonstrated that the antigen’s persistence would cause the tests to stay positive for a minimum of seven days after treatment [28].

The duration of a positive RDT result after recommended treatment is mainly dependent on the density and duration of parasitemia before treatment, as well as the parasite-specific expression of HRP2. In one study, the mean duration of persistent antigenicity in Ugandan children who had a successful antimalarial treatment was 32 days [29]. This duration varied significantly depending on the pre-treatment parasitemia: patients with a parasite density >50,000/μL had, on average, persistent antigenicity for 37 days whereas patients with a density of <1000/μL had, on average, a persistent antigenicity of 26 days [29]. A more recent study looked further into the median half-life of HRP2 in blood by measuring HRP2 levels in individuals from Angola, Tanzania, and Senegal participating in therapeutic efficacy studies [30]. By fitting a first-order kinetics clearance model to the HRP2 concentration versus time (days), median clearance rate constants varied from 0.15 to 0.23 day^−1^ among countries, resulting in median half-lives of 4.5, 4.7, and 3 days in Angola, Tanzania, and Senegal, respectively. Such consistency in HRP2 clearance across different African regions suggests the presence of a common biological mechanism influencing HRP2 dynamics upon resolution of *Pf* infection [30]. It has also been hypothesized that the length of HRP2 positivity partly depends on the presence and affinity of host anti-HRP2 antibodies [28].

Highly sensitive RDTs (hsRDTs) claimed to have a 10-fold increase in sensitivity compared to the RDTs routinely available in the market [31]. Therefore, they provide the opportunity to more efficiently detect cases of malaria with low-parasitic densities and asymptomatic infections [32]. Traditional RDTs have a limit of detection (LOD) of approximately 800 pg/mL and show decreased sensitivity to detect low-density *Pf* infections (<100 parasites/µL). hsRDTs (with a LOD of 80 pg/mL) allow the detection of low-density *Pf* malaria infections (<100 parasites/µL), performing closely to expert microscopy (detection limit of 4–20 parasites/µL) [31]. However, this increased LOD can lead to a rise in the absolute number of false-positive results, as levels of persisting HRP2 that went undetected to date can now be picked up by hsRDTs [31,33]. In fact, a study utilizing participants from Angola, Tanzania, and Senegal countries demonstrated that the median duration of post-treatment RDT positivity increased from 13 to 16 days when utilizing the hsRDTs compared with conventional RDTs [30].

While the persistence of HRP2 can be seen as a drawback for malaria diagnosis and clinical management, it can also be used as a method to assess recent infections in non-infected people and consequently more accurately determine ongoing transmission [34,35,36]. Additionally, researchers have demonstrated that cases of recrudescence have different patterns in HRP2 concentrations over time. A study reported that infections which did not clear parasites due to treatment failure presented higher HRP2 levels at day 3 post-treatment compared with successfully treated infections, which suggests that these early changes in HRP2 levels could be used to monitor treatment success [30]. However, the field implementation of quantitative immunoassays, including the highly sensitive bead-based assay used in the above-mentioned studies [30,35], that allow antigen quantification appears logistically difficult, and therefore novel quantitative tools are needed if HRP2 is intended to be used as a marker for treatment prognosis.

### 2.2. Variability of P. falciparum HRP2 and Homology with HRP3

The *Pf* genome encodes several proteins, including HRP2 and its structural homologue HRP3, that uncommonly contain repetitive sequences comprising a small handful of amino acids [9,37,38]. The mature configuration of HRP2 contains histidine-rich sequences that form the epitopes targeted by the mAbs in RDTs [9,18,39]. Past studies demonstrate that genetic variations (polymorphisms, gene deletions and duplications) have the potential to translate into different protein sequences [40]. HRP2 has been shown to be polymorphic in sequence composition of the repeated motif, leading to more than 30 sequence variants (or types) identified to date [38]. Additionally, there is significant polymorphism in both the number of repeated motifs and length between different parasite strains [9,38]. All considered, the question of whether such extensive diversity in HRP2 potentially affects the sensitivity of RDTs has been introduced, but few studies have since addressed it.

The first study to observe extensive diversity in HRP2/3 sequences sequenced 75 *P. falciparum* lines and field isolates from 19 countries [9]. In this study, they identified a total of 18 unique amino acid repeats within the protein sequences of HRP2 and HRP3. Four of the repeat types (named 1, 2, 4 and 7) are shared by both proteins, repeat types 15–18 are specific for HRP3, and the remaining repeats are only present in HRP2. Of the 14 amino acid repeats identified in HRP2, only 5 were present in all isolates [9]. In addition, they showed variation in the frequency of repeats among different *Pf* isolates, as well as in the total number of repeats, its order within the sequence, and the number of each repeat within HRP2. These variations led them to identify 56 unique HRP2 sequences. Regarding the role such diversity could play on the performance of RDTs, they reported that isolates with higher numbers of type 2 and type 7 repeats were better recognized, particularly at low parasitemias (<250 parasites/µL). Remarkably, it was shown that the laboratory *Pf* D10 clone, lacking the *pfhrp2* gene but positive for *pfhrp3*, could be detected by HRP2-based RDTs, suggesting that the candidate antigen for this cross-reactivity is HRP3. This aligns with a previous study that reported an 85–90% homology in nucleotide sequence-flanking repeats between *pfhrp2* and *pfhrp3* and an amino acid substitution of D/N in the major repeat of AHHAAD/N in a single parasite line [8], as well as with reported data on cross-reactivity between HRP2 and HRP3 for various HRP2-specific monoclonal antibodies [6].

In Madagascar *Pf* isolates, 93% (n = 13/14) of different HRP2 repeats identified in previous studies were detected in 229 successfully amplified *pfhrp2* fragments, while for HRP3 protein, seven out of eight repeats were identified [41]. *pfhrp2* sequence analysis predicted that 9% of Malagasy isolates would not be detected at parasite densities ≤250 parasites/μL [41]. The PfHRP2 and PfHRP3 antigens were found to be highly diverse in parasite isolates throughout Madagascar [41]. A later study identified new repeat sequences when 458 isolates from 38 countries were analyzed. These repeats (19–24) were specific to the HRP2 protein except for type 20, which was also present in the HRP3 protein [40]. Moreover, this study classified amino acid sequences for the HRP2 protein as Type A, B, or C depending on the product of the type 2 (AHHAHHAAD) and type 7 (AHHAAD) epitope repeats. *Pf* HRP2 Type A comprises the higher number of defined epitopes, the part of an antigen molecule to which an antibody attaches itself, followed by Type B and Type C. However, the predictability of RDT sensitivity using type 2 and type 7 repeats was not confirmed, as no correlation between detection rate using RDT and gene structure was identified, especially when the parasite density was <200 parasites/µL [40].

Studies in *Pf* isolates from India [39,42,43,44], the China-Myanmar border area [45], Myanmar [46], Yemen [37], Senegal [47,48,49], Mali [47], Uganda [47], French Guiana [50], Kenya [51,52], Mozambique [53], Tanzania [53], Nigeria [54], Sudan [38], Ethiopia [55], and Colombia [56] identified HRP2 and HRP3 sequences with different frequencies and number of repeats in each geographic area, although this trend was not identified in all cases. For example, repeat types 9, 11, 13, and 14 have rarely been observed in Africa [49]. However, all studies have described some similarities among isolates. One study [39] demonstrated that the amino acid sequences of HRP2 began with the type 1 repeat and ended with the type 12 repeat, and that repeat types 2 and 7 were found to be highly abundant. The same study also found a higher quantity of unique HRP2 protein sequences compared to HRP3 sequences. Furthermore, a study identified five novel repeats within HRP2 in Indian isolates [39]. In this same study, a relationship between the number of type 2 and type 7 repeats and RDT detection rate was statistically confirmed [39]. Similarly, a study in isolates from Senegal reported a mild association between the number of type 2 repeats and poor HRP2 RDT diagnosis (*p* = 0.046). The authors reported an increase in the number of isolates predicted to be non-sensitive to HRP2 RDTs based on HRP2 sequence for the years 2009 to 2011, suggesting the possibility of diagnostic selection pressure at play [49]. On the contrary, another study quantified HRP2 plasma levels by enzyme-linked immunosorbent assay (ELISA) in plasma samples with parasites of diverse pattern of repeat types and did not find a relationship between the number of repeats observed, or overall sequence length and HRP2 plasma concentrations [53]. Consistent with these findings, a study on Indian isolates observed no association between the number of type 2 and 7 repeats and the RDT performance to detect low-density infections [43]. In the same study, analysis of isolates led to the further identification of three and two new repeat sequences within HRP2 and HRP3, respectively, which resulted in a total of 34 repeat sequence variants. A study among Sudanese isolates also revealed that the RDT performance was not affected by a high-level of genetic diversity in HRP2 [38]. Recent studies in Kenyan [51,52] and Indian [44] isolates have also showed the presence of several new repeat sequences within HRP2 and HRP3.

To summarize, the *P. falciparum* antigen HRP2 presents significant variation, but findings regarding the effect of such variability in RDT reactivity are divergent. This discordance among the several studies on the functional relevance of HRP2 repeats indicates that there is no established relationship between the RDT performance in the field and the number of repeats. However, it has been shown that recombinant HRP2 protein Type A is recognized with greater sensitivity in immunoassays compared with Types B and C [35]. Therefore, it is logical to think (but not easy to strongly prove) that some field isolates are more easily detected than others, particularly at low parasite densities.

### 2.3. P. falciparum hrp2 and hrp3 Gene Deletions

*P. falciparum hrp2* and *hrp3* genes do not only undergo extensive polymorphism but are also subject to deletions. Increasing false-negative HRP2-based RDT reports have rendered concerns, which have in turn led to the need for inclusion of other assays in malaria case management and elimination programs [10,11]. The first report on the presence of *Pf* isolates with *pfhrp2* gene deletion came in 2010 from Peru [57], where eight among nine isolates collected in 2007 lacked the *pfhrp2* gene and six lacked both *pfhrp2* and *pfhrp3* genes when tested by PCR. Plasma HRP2 levels were also not detectable by ELISA [57]. A retrospective analysis of 148 *Pf* isolates collected between 2003 and 2007 revealed that 41 and 70% of the isolates presented *pfhrp2* and *pfhrp3* gene deletions, respectively. In addition, 22% of the isolates lacked both *pfhrp2* and *pfhrp3* genes [57]. Another study from Peru also revealed that 20% of *Pf* isolates collected between 1998 and 2001 had a deleted *pfhrp2* gene [58]. Increased *pfhrp2* deleted *Pf* isolates (40%) were observed in samples collected from Peru between 2003 and 2005, suggesting that *pfhrp2* deletion has occurred many times in the *Pf* isolates collected from this country [58].

**Figure 1:**
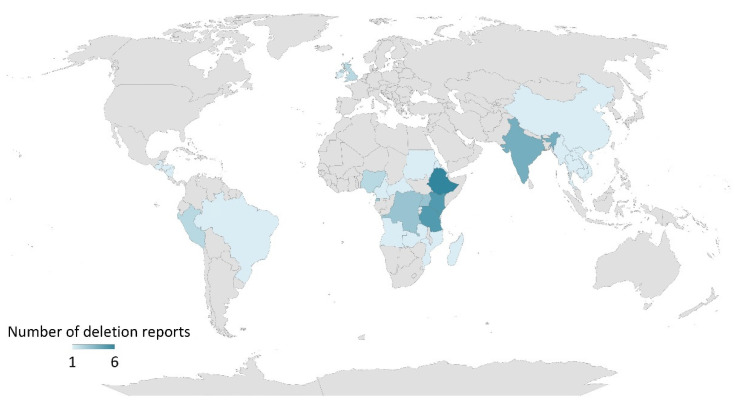
Reports on *pfhrp2/3* deletions by country, 2018–2022. The map was created using Microsoft Excel.

The occurrence of *pfhrp2* deletions is not just confined to South America but has also affected malaria endemic regions in Africa and Asia [10,11]. In 2010–2017, published reports of *pfhrp2/3* deletions came from several countries in Africa (Mali, Mozambique, Senegal, Ghana, Kenya, Democratic Republic of Congo, Rwanda, Eritrea), Asia (India, China-Myanmar border), South America (Peru, Colombia, Brazil, French Guiana, Ecuador, Guyana, Suriname, Bolivia) and Central America (Honduras), which have been previously reviewed [10,11]. In 2018–2022, more reports from countries of African, Asian, South American and North American continents, as well as reports on travelers from the United Kingdom and Ireland, presented data on *pfhrp2/3* deletions (Figure 1, Supplementary Table S1). These studies were identified in PubMed using the following search terms: (‘pfhrp2′, ‘pfhrp3′, and ‘deletion’) or (‘hrp2′, ‘hrp3′, and ‘deletion’) and publication dates from 01 January 2018 until the time of writing (14 July 2022). Based on these published reports, it can be concluded that *Pf* isolates with one or both *pfhrp2/3* gene deletions are circulating throughout the world with varying prevalence in both high- and low-transmission areas. This scenario could adversely impact the life of an affected individual due to health consequences from delayed treatment or no treatment, while also affecting overall healthcare efforts in malaria case management and preventing ongoing transmission.

The continuous use of HRP2-based RDTs in malaria endemic areas (where *pfhrp2/3* deletions are present) can lead to numerous negative consequences in public health. Thus, the WHO should endorse synchronized tactics to examine, map, and report *pfhrp2/3* gene deletions through the provision of standardized protocols and procedures. At present, there are a few traditional methods to determine *pfhrp2/3* deletions including PCR, PCR followed by DNA sequencing, and ELISA-based assays [9,57,59]. However, these methods have several limitations in terms of test sensitivity and the ability to discriminate between multiple clones. Furthermore, the WHO should identify reference laboratories that can provide full or partial support in *pfhrp2/3* gene deletion analysis and its validation, as well as laboratories that can perform complementary serological assays. A common approach will have a huge impact on malaria diagnosis using RDTs in the field as well as on future policy development.

Targeted deep sequencing of important genes could also be an alternative approach to identify genetic variations [60]. Advances in how genetic material is sequenced using next-generation sequencing (NGS) technology have moved genomics from the bench to the field. The rapid acquisition of millions of short nucleotide sequences through NGS provides a high-throughput approach to quantify the frequency of particular *Pf* genetic variants. The copy number variations (CNVs) such as gene deletions and duplications can be determined based on the mean depth of coverage of individual amplicons between the reference sample (3D7, without CNVs) and test sample. This approach can also be combined with other aspects of malaria elimination activities including capturing low-frequency resistant mutants [60,61]. Moreover, combining NGS with pooling of individual isolates can potentially provide a faster and cheaper surveillance tool at the population level [61]. Studies utilizing NGS technology have successfully reported *pfhrp2/3* deletions [62,63]. Another potential alternative for population analysis of *pfhrp2 and pfhrp3* deletions lies in the screening of large sample sets by multi-antigen detection, particularly in the WHO African Region, where *Pf* infections are dominant. The recent development of highly sensitive laboratory-based assays that detect pLDH [64,65] or pLDH and pAldo [66] in combination with HRP2 offer the possibility to predict *pfhrp2* deletions in samples in which pLDH and/or pAldo are positive, but HRP2 is not. However, such an approach would need to be accompanied by further testing with molecular assays to confirm the gene deletions. Rapid and efficient methods to assess diagnostic failure trends can help to guide the most useful and efficient forms of malaria case management. Such surveillance is critical during malaria elimination activities, when mass drug administration is rolled out in the community and fast detection of malaria parasites is crucial.

## 3. Host Factors

### 3.1. Host Immunity

Host factors, including inherent and acquired immunity, age, and pregnancy, play an important role in the performance of HRP2-based RDTs, specifically their influences on detection of low-density infections and with comorbidities. The first study (1986) to produce antibodies against HRP2 immunized BALB/c mice with the *Pf* Malayan Camp strain [67], and since then, several antibodies (both polyclonal and monoclonal) have been generated in mice or rabbits for research purposes and to support the overall development of HRP2-based RDTs. Some years later, another study immunized *Aotus* monkeys with a fusion protein that contained the repeat Ala-His-His sequence and had high homology to HRP2 [68]. After being challenged with *Pf*, immunized monkeys exhibited lower parasitemias compared with control monkeys, suggesting that the antigen was able to trigger antibodies production and confer protection [68,69]. Therefore, one can assume that *Pf*-infected people produce anti-HRP2 antibodies, which raises the question of whether these antibodies would interfere with HRP2-detecting immunoassays [70]. Nevertheless, antibody responses against HRP2 in humans have been scarcely studied.

### 3.2. Patient Age and RDT Performance

While the persistence of HRP2 has been explored to test the sensitivity and function of RDTs, it also provides valuable insight into the relationship between patient age and acquired immunity. Researchers categorized RDT-related papers to demonstrate the average persistence of HRP2 and pLDH by 1) patient age, 2) RDT type, and 3) treatment received, which included artemisinin-based combination therapy (ACT) or non-ACT. They then utilized a Bayesian survival model to estimate the time needed for an RDT-positive patient to turn RDT-negative after treatment. In total, 67 individual study groups were included in the analysis. Through modeling, the study illustrated that HRP2 persistence is more prevalent in children than adults, and individuals treated with ACT have more persistent positivity rates compared to those with non-ACT. In addition, persistent positivity is predicted for a longer duration when using HRP2 RDTs than when using pLDH-only or HRP2/pLDH combination RDTs [22].

Among global malaria deaths, children under the age of five face the greatest threat from the malaria parasite. In areas of differing malaria transmission, the threat is even higher, as RDTs fluctuate in specificity and sensitivity in comparison to the “gold standard” of malaria microscopy [71]. The WHO recommends that RDTs should perform at a minimum sensitivity of 95% and a minimum specificity of 90% [21]. RDTs with sensitivity and specificity below the satisfactory limits should be used with caution [72,73]. A cross-sectional survey in southern Tanzania compared the performance of *Pf* RDTs across differing age groups and transmission intensities [71]. They found that test sensitivity was high (ranging from 98 to 100%) among participants below 25 years but decreased to 81.3% in older adults. In addition, the test specificity varied dramatically across age groups, including a drop to 25% in children of ages 5–9 [71]. An additional study in Kenya found that RDTs used on children ages 9–14 living in hyperendemic transmission settings had an average of 87% sensitivity and 88% specificity, both percentages are under the WHO’s recommended baseline of RDT diagnostic requirements [74]. With worsened RDT performance in the most vulnerable age group, it is necessary to take caution when utilizing RDTs on children and young adults, as positive results can be missed due to lower specificities and sensitivities of the tool.

### 3.3. Performance of RDTs on Pregnant Women

Research exploring the relationship between pregnancy and malaria has increased, especially in identifying the accuracy and limitations of RDT use on pregnant mothers [75]. Malaria during pregnancy can result in many neonatal complications such as premature delivery, low birth weight, congenital malaria, or even neonatal death [76,77]. Additionally, pregnant mothers are at a higher risk of developing severe anemia and have higher rates of maternal death [76,77]. In a study conducted in Burkina Faso and Uganda, researchers compared the use of HRP2 and pLDH-based RDTs, expert microscopy, and PCR for detection of malaria during pregnancy and at delivery [78]. The HRP2-based RDT appeared to show the highest performance rate in detecting *Pf* among the testing mechanisms as the RDTs were able to detect HRP2 in the placenta (due to the parasite’s characteristic sequestration), as well as in maternal peripheral blood (due to the antigen’s high persistence) [78]. Although the sequestration of the parasite in the placenta allows for better detection of malaria in pregnant mothers as the parasite would otherwise be undetected in a standard smear, it drastically increases the chance of negative neonatal and maternal health outcomes [75,76].

Intermittent preventive treatment of malaria in pregnancy with sulfadoxine-pyrimethamine (IPTp-SP) has been a tool to reduce the incidence of maternal anemia, low birth weight, and neonatal mortality in pregnancy from malaria [79]. However, the development of parasitic resistance to the SP drug, especially in parts of Africa, has severely impacted the success of IPTp-SP [80]. Remarkably, trials in Africa that administered intermittent screening and treatment in pregnancy (ISTp) alongside dihydroartemisinin—piperaquine to positive malaria cases identified this treatment pairing as potentially feasible [81,82,83,84,85,86]. However, the frequency of missed diagnosis by RDTs for malaria raised concern [87]. The process involved intermittent screening of pregnant women in their second and third trimesters using RDTs and then treating HRP2-positive women with antimalarials [83,87]. In one study, primigravidae and secundigravida mothers in four West African countries (Ghana, Burkina Faso, The Gambia, and Mali) participated in an ISTp trial in which they were screened by HRP2/pLDH RDTs on enrollment and during antenatal clinic (ANC) visits [87]. The study aimed to identify RDT sensitivity levels and evaluate malaria detection rates throughout pregnancy. Results showed that RDT sensitivity remained high when detecting malaria in primigravidae and secundigravidae at enrollment in three of four countries. In Ghana, RDT sensitivity was 89, 83 and 77% at enrollment, second and third ANC visits, respectively, but only 49% at delivery. Researchers proposed that the drop in RDT sensitivity was due to a substantial drop in parasite density at delivery compared with enrollment because of earlier treatment of RDT-positive infections as well as development of immunity as the pregnancy developed [87]. Additional studies have also demonstrated high sensitivity levels of RDTs in this screening process [75,88]. The use of RDTs throughout ISTp treatment is a potential step toward improving malaria case management for pregnant mothers, although caution should be taken to ensure that the sensitivity levels of RDTs used are adequate to properly detect the parasite.

Furthermore, the difference in RDT sensitivities still raises concerns for the number of asymptomatic and low-density infections that are not identified by the RDT. Researchers in Colombia compared the performance of hsRDTs, light microscopy (LM), and conventional rapid diagnostic tests (Pf/Pv RDT and Pf RDT) on 737 peripheral and placental specimens collected from pregnant women. The improved sensitivity of the hsRDT resulted in better detection of HRP2 in comparison with both LM and conventional RDTs [89]. Another study also demonstrated a higher hsRDTs sensitivity compared with microscopy and conventional RDTs [90]. However, in Indonesian women, no significant differences were found between the performance of hsRDTs and conventional RDTs [91]. Overall, the creation of hsRDTs shows a promising future for its implementation as a reliable testing tool, although further research should explore the effectiveness of this tool, especially in patients with low-density infections and in low-transmission settings.

### 3.4. False-Negatives, False-Positives, and Patients with Co-Infections

Since each infected person experiences differing levels of parasitemia, symptoms, and immunological responses, RDTs are placed at a disadvantage to provide an accurate reading for each individual case. As excess parasite antigens bind with antibodies without available epitopes for capture, commonly referred to as the test-band antibody, the high parasite density can lead to the prozone effect, also referred to as the high-doses hook phenomenon, which occurs when HRP2 is unable to be detected by the RDT [92,93]. This effect is frequently cited as a cause of false-negative RDT results [92], although it lacks extensive documentation in scientific papers. One study worked to bridge the gap between the lack of prozone effect documentation by testing 22 different RDT brands with various levels of clinical samples of *P. falciparum* hyperparasitemia (>5% infected red blood cells) [92]. Each of the 22 RDT brands were tested with seven samples, both undiluted and upon 10×, 50× and 100× dilution in NaCl 0.9%. Of the 17 HRP2-based RDT brands, 16 were affected by the prozone effect in 6/7 samples. The prozone effect was confirmed by researchers when there was an increase in test line intensity of at least one category after dilution (negative, faint, weak, medium, or strong), and when observed by two readers through duplicate testing. Pf-pLDH tests did not demonstrate the prozone effect for any of the tests [92]. A similar study conducted in Mozambique reported comparable results. With almost identical testing methods, all six of the HRP2-based RDTs demonstrated the prozone effect while zero of the Pf-pLDH tests scored positive for the effect [94]. These studies provide evidence that the prozone effect is indeed a cause of false-negatives of HRP-2 RDTs in blood samples with high parasitic densities. In addition, the prozone effect oftentimes results in negative consequences for a patient as their symptoms can be misdiagnosed for a different infection [95].

Issues with RDT diagnosing continues with the increase in complexities surrounding asymptomatic malaria (AM) cases in different levels of endemicity. AM is defined by the lack of fever or other identifying clinical symptoms, which oftentimes results in infected individuals not seeking treatment. Meanwhile, these asymptomatic cases are missed by passive surveillance while remaining an important gametocyte reservoir and contributing to the persistence of malaria transmission [96]. Individuals with AM can also develop exposure-dependent immunity that partially protects against future illness/severity, which is an obvious benefit, although high numbers of parasites can remain in the blood [96]. On the other hand, AM cases have been associated with recurrent episodes of symptomatic parasitemia, chronic anemia, maternal and neonatal mortality, co-infection with invasive bacterial disease, cognitive impairment, and continuous transmission [97]. Additionally, as AM infections are often accompanied with low-density parasitemia, these individuals are likely to remain undiagnosed during RDT testing [95,96], subsequently becoming potential reservoirs for disease transmission, and thus hampering malaria elimination efforts.

The performance of RDTs is hindered when it comes to diagnosing malaria patients with co-infections. In some cases, malaria patients with a co-infection can present as RDT positive, but only exhibit symptoms due to that second infection [95]. Although the RDT can accurately identify the presence of HRP2 in patients, it fails to pinpoint the cause of the symptoms. This shadowing of symptoms can lead to unsuccessful treatment of the second infection, as patients are treated for malaria instead of the infection causing the symptoms. Such instances have been reported in patients experiencing a malaria co-infection with the hepatitis C virus, Dengue virus, or infection from *Toxoplasma gondii* (reviewed in [95]). Further research to explore the impact of comorbidities, the prozone effect, and AM on RDT performance would greatly improve malaria case management.

## 4. Environmental Factors

### 4.1. Low-Transmission Settings

The changing epidemiology of malaria, especially in low-transmission settings presents new challenges for malaria diagnosis and case management. In countries with low transmission, a higher proportion of asymptomatic infections are below the reliable detection limit of microscopy [98] and RDT [99]. This proportion of asymptomatic infections may depend on how fast malaria transmission wanes, with a significant reservoir of asymptomatic carriers expected when the decrease in transmission is faster than the loss of immunity in a population [96]. On the contrary, the asymptomatic reservoir would be minimal when the transmission has decreased slowly over many years and people have lost all antimalarial immunity [96].

The poor sensitivity of RDTs in low-density infections raises great concern on the performance of RDTs in areas of low malaria transmission [100]. A study in Swaziland, a low-endemicity country aiming for malaria elimination, tested patients suspected of having malaria throughout 37 health facilities to evaluate the accuracy of RDTs in low-transmission settings [100]. Their results demonstrated a low RDT sensitivity during testing, which they hypothesized was due to the large proportion of low-density infections in subjects enrolled in the study [100]. Furthermore, another study in Rwanda compared the sensitivity of HRP2 and pLDH RDTs to microscopy (thick smears) during a time of decreasing transmission intensity in the region of Kibirizi [101]. Researchers found that during this drop in transmission intensity, the slide positivity dropped from 46 to 3%. Additionally, they found that the decline was associated with a decrease in RDT sensitivity (HRP2-based) from 88 to 67% [101]. This drop in sensitivity supports the evidence that low-transmission levels correlate to lower RDT sensitivity [99]. However, studies have also demonstrated that RDTs can be a useful tool to detect malaria in a low-transmission setting [14,102]. Steps should be taken to ensure that patients are accurately diagnosed as missed diagnosis of patients who are positive with the malarial infection can result in a failure to properly treat the patient as well as can hinder the efforts toward elimination.

### 4.2. High-Transmission Settings

The performance of HRP2–based RDTs to detect *Pf* infection in moderate-to-high-transmission settings is shown to be reliable [103,104]. With this said, RDTs used in these transmission intensities still possess limited reliability when detecting lower parasitic densities [105]. In a 2014 study conducted in a hyperendemic region of Burkina Faso, the performance of RDTs and microscopy was compared in asymptomatic carriers. The researchers found that after three community screenings of asymptomatic carriers, the sensitivity of the HRP2-based RDTs decreased (92.4% compared to 77.8%) with each of the three screening campaigns [105]. Additionally, it was found that across all age groups, the sensitivity of the RDT increased alongside an increase in parasitic density. Likewise, false-negative rates were the greatest in the final campaign, showing that a decrease in the parasitic reservoir leads to a decrease in RDT performance. They concluded that one way to reduce the incidence of false-negatives is by using a loop-mediated isothermal amplification in combination with an HRP2 test, as long as the resources for testing are available in the community [105].

High-transmission areas signify a high prevalence of parasitemia in the population and lead to frequent malarial infections, the majority being in children under the age of five [106]. A study in Uganda was conducted to assess the RDT use in improving fever management in areas of high transmission [106]. Sensitivity remained high for both HRP2 and pLDH RDTs throughout the study but dropped drastically for HRP2 in terms of specificity for patients with recurrent fever episodes. The HRP2 RDT also had 51% false-positives by day 28 of the study, whereas the pLDH RDT gave no false-positives after day 7 [106]. In this case, researchers recommended using pLDH-based RDTs for monitoring and diagnosing recurrent malaria episodes in a community, as sensitivity and specificity did not have any sudden drops throughout the trial [106]. In addition, another study’s results suggested that HRP-2 test performance in areas of intense malaria transmission can also be influenced by age and the prevalence of *Pf* infection. In children, low specificity can cause over-estimation of malaria prevalence [71]. Overall, caution should be taken when using RDTs in high-transmission settings when there is a documented decrease in parasitic density, as false-negatives and false-positives can lead to poor case management and death.

### 4.3. Transmission and Weather Patterns

Transmission rates are shown to directly correlate with weather patterns, as rates of malaria infection are near zero during the end of the dry season [107]. A study tracked patterns of malaria infection to assess the relationship between seasonal resurgences and the Sahelian environment [107]. In high-transmission periods (including the wet season), the ratio of clinical to asymptomatic malaria cases was roughly 0.5. On the other hand, this ratio increased fivefold during the low transmission periods [107]. In the dry season, there was an association between stable malaria transmission and sustained asymptomatic carriage. These results suggest that control strategies should be implemented to target continual low-level parasitemia clusters and halt residual transmission [107].

A study conducted in Burkina Faso tested the sensitivity and specificity of RDTs in both the rainy and dry seasons [108]. Researchers documented that 28.3% of patients were RDT-positive fever cases in the dry season with RDT sensitivity and specificity measured at 86 and 90%, respectively. In contrast, while 68.2% of RDT-positive patients were identified in the wet season, the RDT sensitivity and specificity were 94 and 78%, respectively. These results show that although there are fewer cases of malaria during the dry season, the low parasitic counts during the dry season may cause a decrease in RDT testing sensitivity [108]. For now, it is very well recognized that for active detection of asymptomatic cases, RDT performance is limited [109,110].

## 5. Additional Factors

### 5.1. Handler and Operation Error

Apart from the biological factors affecting testing accuracy, RDT performance is also greatly impacted by human error. Faulty handling, improper storage, and poor operation are all limitations of using RDTs in the field, especially in communities with few resources and training programs [111,112]. For some areas, RDT testing is completed by community health workers (CHWs), individuals who play an important role in ensuring resource-scarce communities receive quality care. To learn how to use the RDT, these workers receive training by a more experienced professional (such as a doctor or researcher) or simply rely on printed instructions from the RDT manufacturer. One study measured the effect of extra instruction by health workers on the use of RDTs to see if the additional instruction improved the accuracy of testing compared with the sole use of the manufacturer’s guide [113]. In the Philippines, it was demonstrated that the correct use of the RDT dipstick and cassette increased by 17% [113] after this additional instruction. Unfortunately, errors in the administration and interpretation of the results were still common among testers. Furthermore, researchers noted that an additional training on proper use and interpretation improved accuracy, from 70 to 80% [113], demonstrating the need for in-person training over sole reliance on the manufacturer’s guide. Another study also suggested that well-designed instructions plus training of CHW’s can ensure the high performance of RDTs [114]. In Burkina Faso, CHWs could perform RDTs with acceptable sensitivity and specificity [115]. Although simple in design, RDT testing requires in-depth training, clear instructions, and long-term monitoring to ensure accurate testing outcomes for the community.

In 2015, the WHO conducted a large-scale product examination to test the performance of RDTs on the market, which included testing of 46 products in the categories of panel detection scores, false-positive scores, heat stability, ease-of-use description, labeling and ease of instructions [116]. For the final category, technicians rated if the RDT components were correctly labelled (box, cassette, buffer container, accessories) and if the instructions were easy to navigate. The study found that although the majority of RDTs showed good adherence to the labeling guidelines of the main package, many lacked important warning labels, such as those stating if the product was single use or advising to dispose of lancets in the sharps container [116]. The lack of these important warning labels raises great concern for the health and safety of both the user and the patient, as needle pricks with malaria-infected blood could spread the parasite. Such an instance was noted in a case with a French nurse, who tested positive for *P. vivax* after a needlestick injury in 2001 [117]. Additionally, less than 50% of the products advised the user to consider a faint line on the RDT a positive result, which leads to many missed diagnoses in patients [116]. Other issues included a lack of information on parasite densities, a failure to include limitations of the product, or a failure to add references for other methods [116]. These findings suggest that for RDT users to conduct proper, more accurate testing, steps must be taken from the production standpoint to promote clarity between the testing device and the user.

### 5.2. Inadequate Storage: Effects of Heat and Humidity

Due to the sensitivity of the testing device and rural tropical locations (with varying temperatures) where testing is mainly conducted, RDT performance is widely affected by improper storage. Excess exposure to heat and humidity goes hand-in-hand with limited resources and training in a given area. Communities with low resources are less likely to have the facilities and equipment to keep RDTs in the recommended storage environment, leading to false readings [118]. Most manufacturers recommend RDT storage between 4 °C to 30 °C but during transportation, RDTs get exposed to excess heat and humidity in rural tropical zones [118,119]. A study demonstrating the effects of heat on pLDH and HRP2 detecting RDTs illustrated the need for proper storage to improve accurate testing outcomes [120]. Researchers incubated the RDTs at 35, 45, and 60 °C at which pLDH RDTs showed an exponential fall in test line positivity as heat increased [120]. Paired with high temperatures, humidity accelerates denaturation, leading many manufacturers to protect the test strips in hermetically sealed packets with a desiccant, a substance that induces a state of dryness and prevents exposure to excess humidity [121]. The exposure of RDTs to a wide range of temperatures was documented during the course of travel to field laboratories in the Philippines and Cambodia [118]. For both countries, storage and transportation temperatures frequently exceeded the lower and upper limits for most RDTs [118]. An additional study utilized monitoring devices to document temperatures and humidity levels of RDT storage facilities in Burkina Faso, Senegal, the Philippines, and Ethiopia over a 13-month period. Their results showed that the temperature regularly exceeded 30 °C, and that the maximum humidity limit was above 94% for all four countries [119]. These studies verify the common influx of temperature exposure during travel, revealing the need for efficient, controlled transportation and storage methods for RDTs in the field.

A way to prevent denaturation and ensure functionality of the RDT is through the establishment and maintenance of a cool chain. By definition, a cool chain means ensuring an adequate temperature of the RDT through all levels of production and delivery, including stages of shipping, transportation, and storage [122]. The cool chain begins with the shipment from the manufacturers, in which the consignee should verify flight details, confirm the shipping notification, and place orders for the shipment to be held in a temperature-controlled location onsite [122]. The next step, ground transportation, must be promptly carried out. During this time, the RDT should not be placed in direct sunlight, especially when the vehicle is parked. The final step of the cool chain, storage, requires RDTs to be placed in shaded, centralized, and controlled locations. It is recommended that RDTs are stored in locations with thatched roofing over iron roofing and in places with evaporative cooling cabinets when possible. Overall, monitoring the RDT sensitivity during intervals of use should be conducted to ensure the functionality and safety of the tool.

### 5.3. Accessibility and Acceptance of RDTs in a Community-Based Setting

As delivery of care evolves, it is necessary to understand how the accessibility and acceptance of RDTs in a community can change depending on the current political, social, and economic status of a country. Currently, there are three prime modes of delivery for malaria care across the globe. These include public health care (sponsored by the government), the private sector (formal or informal), and care distributed by CHWs [123]. While the public health sector can treat low-income individuals, resources are oftentimes not able to reach those living in remote locations, and the burden of disease falls even harder on vulnerable populations, such as women and children. Therefore, the duty falls on CHWs and their use of RDTs to properly diagnose and treat malaria cases in these sparsely populated regions. According to the WHO, sub-Saharan Africa has the fewest number of doctors yet maintains the highest level of malaria cases worldwide, with children under five accounting for 80% of malaria deaths [3]. In these low-resource countries with few doctors, alternative methods of malaria testing must be increased to improve case management [124]. In the text, “Optimizing Malaria Treatment in the Community,” the authors provide key recommendations for CHWs working in low-resource settings. With a focus on education and long-term monitoring of testing methods, testing completed by CHWs has shown an increase in both the accuracy of RDT testing procedures and interpretation of results [114,124,125].

In Angola, the largest portion of healthcare demands is due to malaria, and differences in resources for each province lead to healthcare inequalities across the country [126]. A health facility survey was performed across 89 locations in two provinces of the country to determine differences in facility readiness and RDT availability. These provinces included Huambo, a region of stable endemicity, and Uíge, a region of hyperendemicity. The results showed that, on average, 71% of health facilities in Huambo had ready access to RDTs or microscopy compared to 85% in Uíge [126]. After evaluation of the care-management pathways of each province, it was found that 18 (25%) of true malaria cases were managed correctly in Huambo while 130 (56%) of all true cases were managed correctly in Uíge [126]. Researchers did not find an association between RDT readiness and case management, although the testing step was determined to be the main determinant of overall case management quality [126]. These less than ideal percentages regarding RDT readiness accompanied with an unequal success rate of malaria case management demonstrate the need for improved access to resources across the country, especially in the category of training among healthcare providers.

Another restraint to RDT testing is the acceptability of the diagnostic tool within the population [124]. In a 2010 study in the Uganda’s Iganga district, acceptability levels toward using RDTs were evaluated by conducting focus group discussions with community-trusted community medicine distributors, who mimic the role of CHWs in the district [127]. In these discussions, community members expressed fear that the RDT testing procedure could leave them and their community vulnerable, including fears of HIV infection, or that the samples would be used for witchcraft [127]. Even when RDTs are adopted by a community, there is still a percentage of the population who does not believe in the accuracy of the results. Such was the case in a 2009 study in Sudan, in which CHWs faced difficulties persuading 30% of volunteers that their negative RDT results were genuinely negative [128]. Lastly, distrust of CHWs, especially in regions where resources are extremely low, prevents patients from coming to health facilities as they face rumors of out-of-stock testing tools and do not want to waste personal resources to travel to a facility that is not able to provide diagnosis and treatment [124]. This accumulation of factors can prevent patients from seeking and receiving the care they need. Thus, these factors should be considered when developing community care programs for malaria testing and distributing resources to low-income areas. Educational campaigns, especially those debunking the heightened possibility of HIV infection through RDT testing, should be implemented where possible to increase the testing outcomes in a community. Lastly, supply management should be maintained to ensure constant access to RDTs health facilities, which would improve perceptions of the facilities in a community and lead to increased testing.

## 6. Conclusions

As the battle toward eliminating malaria continues, the need to understand and engage in the prevention of factors that hinder testing performance of RDTs becomes crucial. While these tools are facing a series of limitations, both with the testing device itself and the country of testing, their noted success in the field compared to previous diagnostic methods shows great promise in the detection and management of malaria cases around the globe. Rising issues, including the discovery of gene deletions and repeat sequences, require further work to identify where these genetic changes are occurring and expected to occur in future years. More historic issues, such as HRP2 persistence, problems diagnosing asymptomatic malaria cases, and limitations of RDTs in testing pregnant women, children, and patients with co-infections, continually contribute to a significant number of false-positive and false-negative results. Additionally, overarching issues regarding community access to resources, proper training, and acceptability of the diagnostic tool leave room for RDT testing improvement to be tackled with a public health lens.

Regardless of their shortcomings, RDTs are the unsung hero of malaria diagnosing, and with advances in the issues discussed, can continue to be the most effective and leading tool for malaria diagnosis worldwide. With the current price of less than a dollar per RDT, this tool proves to be a cost-effective and viable option for low resource communities, especially in comparison with the other resource-intensive testing methods of a microscopy blood smear or PCR [129]. Additionally, they give results in 20 min, which is phenomenal for delivering instant, same-day results for patients who oftentimes need to travel far to receive adequate testing and treatment. With proper training, CHWs can use RDTs, which increases the number of people who can conduct malaria testing. This is especially effective in low resource settings where hospitals or other healthcare facilities are not always available. Lastly, RDTs have shown promising results for malaria screenings on pregnant women. With RDT testing implemented throughout the pregnancy, proper treatment can be distributed sooner and more effectively.

Overall, RDTs are an effective tool for diagnosing malaria, especially in areas where resources are limited. They are cheap, user friendly, and provide testing results in a timely matter without the need for extensive laboratories or equipment. Previously, an ideal RDT was described as one that “would detect and differentiate between all human malaria species; distinguish low, medium and high parasitemia’s; be available in a temperature-stable format; have internal controls for antigens; be easy to use; produce an unambiguous result; and remain cheap” [21]. Although the ideal RDT does not yet exist, promising work has been done to promote the development of a more inclusive testing device. For example, the creation of hsRDTs allows for increased sensitivity of the tool and heightened detection of HRP2 in low-parasitic densities, which may have the potential to prevent malaria-related deaths and unnecessary treatments. Additionally, the use of mathematical modeling shows great promise in predicting the location of future *pfhrp2* and *pfhrp3* deletions, especially in Sub-Saharan Africa where malaria is most endemic [130]. The identification of these gene deletions suggests the need for more effective, region-based tools that can diagnose malaria without the need to identify HRP2, aiding in cases involving the prozone effect, low-parasitic densities, and sequestration. Lastly, an increase in education, awareness, and training programs will drastically improve the accuracy of RDT use in a community-based setting, the starting line for continual progress toward worldwide malaria elimination.

## Supplementary Materials

The following supporting information can be downloaded at: https://www.mdpi.com/article/10.3390/tropicalmed7100265/s1, Table S1: Reports on *pfhrp2/3* deletions between 2018 to 2022 from different countries. References [131,132,133,134,135,136,137,138,139,140,141,142,143,144,145,146,147,148,149,150,151,152,153,154,155,156,157,158,159,160,161] are cited in the supplementary materials.
