# Supplementary material for: Factors Affecting the Performance of HRP2-Based Malaria Rapid Diagnostic Tests
Source: Trop Med Infect Dis. 2022 Sep 25;7(10):265. doi: 10.3390/tropicalmed7100265 (PMC9611031; doi:10.3390/tropicalmed7100265)
Supplement: Supplementary file 1 [file tropicalmed-07-00265-s001.zip › tropicalmed-1900141-supplementary.pdf]

Supplementary Table S1: Reports on *pfhrp2/3* deletions between 2018 to 2022 years from different countries.

Publication year	References	Country	Continent
2019	Plucinski et al [1]	Angola	Africa
2022	Eyong et al. [2]	Cameroon	
2021	Bylicka-Szczepanowska et al. [3]	Central African Republic	
2021	Parr et al [4]	Democratic Republic of Congo (DRC)	
2021	McCaffery et al [5]		
2022	Nundu et al [6]		
2020	Iriart et al [7]	Djibouti	
2019	Schindler et al [8]	Equatorial Guinea	
2020	Berzosa et al [9]		
2022	Hosch et al [10]		
2018	Berhane et al [11]	Eritrea	
2019	Girma et al [12]	Ethiopia	
2020	Golassa et al [13]		
2021	Feleke et al [14]		
2021	Alemayehu et al [15]		
2022	Rogier et al [16]		
2022	Leonard et al. [17]		
2018	Nderu et al [18]	Kenya	
2018	Sepulveda et al [19]		
2019	Nderu et al [20]		
2020	Grignard et al [21]		
2022	Rogier et al [16]		
2022	Rogier et al [16]	Madagascar	
2020	Galatas et al [22]	Mozambique	
2019	Funwei et al [23]	Nigeria	
2021	Prosser et al [24]		
2022	Rogier et al [16]	Rwanda	
2021	Prosser et al [24]	Sudan	
2019	Thomson et al [25]	Tanzania	
2019	Schindler et al [8]		
2020	Bakari et al. [26]		
2020	Grignard et al [21]		
2022	Kaaya et al [27]		
2019	Thomson et al [25]	Uganda	
2020	Agaba et al [28]		
2021	Nsobya et al [29]		

2019	Kobayashi et al [30]	Zambia	
2020	Gibbons et al [31]	China-Myanmar boarder area	Asia
2018	Pati et al [32]	India	
2018	Sepulveda et al [19]		
2021	Kumari et al [33]		
2022	Kumari et al [34]		
2018	Sepulveda et al [19]	Cambodia	
2018	Sepulveda et al [19]	Laos	
2018	Sepulveda et al [19]	Thailand	
2018	Sepulveda et al [19]	Vietnam	
2018	Sepulveda et al [19]	Peru	South America
2019	Schindler et al [8]		
2020	Goes et al [35]	Brazilian Amazon	
2018	Fontecha et al [36]	Honduras	North America
2018	Fontecha et al [36]	Guatemala	
2018	Fontecha et al [36]	Nicaragua	
2020	Grignard et al [21]	UK travellers	Europe
2021	Nolder et al [37]		
2021	Nolder et al [37]	Ireland travellers	

References:

1. Plucinski, M.M.; Herman, C.; Jones, S.; Dimbu, R.; Fortes, F.; Ljolje, D.; Lucchi, N.; Murphy, S.C.; Smith, N.T.; Cruz, K.R.; et al. Screening for Pfhrp2/3-Deleted Plasmodium falciparum, Non-falciparum, and Low-Density Malaria Infections by a Multiplex Antigen Assay. *J Infect Dis* **2019**, *219*, 437-447, doi:10.1093/infdis/jiy525.
2. Eyong, E.M.; Etutu, S.J.M.; Jerome, F.C.; Nyasa, R.B.; Kwenti, T.E.; Moyeh, M.N. Plasmodium falciparum histidine-rich protein 2 and 3 gene deletion in the Mount Cameroon region. *IJID Reg* **2022**, *3*, 300-307, doi:10.1016/j.ijregi.2022.05.006.
3. Bylicka-Szczepanowska, E.; Korzeniewski, K.; Lass, A. Prevalence of Plasmodium spp. in symptomatic BaAka Pygmies inhabiting the rural Dzanga Sangha region of the Central African Republic. *Ann Agric Environ Med* **2021**, *28*, 483-490, doi:10.26444/aaem/141872.
4. Parr, J.B.; Kieto, E.; Phanzu, F.; Mansiangi, P.; Mwandagalirwa, K.; Mvuama, N.; Landela, A.; Atibu, J.; Efundu, S.U.; Olenga, J.W.; et al. Analysis of false-negative rapid diagnostic tests for symptomatic malaria in the Democratic Republic of the Congo. *Sci Rep* **2021**, *11*, 6495, doi:10.1038/s41598-021-85913-z.
5. McCaffery, J.N.; Nace, D.; Herman, C.; Singh, B.; Sompwe, E.M.; Nkoli, P.M.; Ngoyi, D.M.; Kahunu, G.M.; Halsey, E.S.; Rogier, E. Plasmodium falciparum pfhrp2 and pfhrp3 gene deletions among patients in the DRC enrolled from 2017 to 2018. *Sci Rep* **2021**, *11*, 22979, doi:10.1038/s41598-021-02452-3.
6. Nundu, S.S.; Arima, H.; Simpson, S.V.; Chitama, B.A.; Munyeku, Y.B.; Muyembe, J.J.; Mita, T.; Ahuka, S.; Culleton, R.; Yamamoto, T. Low prevalence of Plasmodium falciparum parasites lacking pfhrp2/3 genes among asymptomatic and symptomatic school-age children in Kinshasa, Democratic Republic of Congo. *Malar J* **2022**, *21*, 126, doi:10.1186/s12936-022-04153-2.
7. Iriart, X.; Menard, S.; Chauvin, P.; Mohamed, H.S.; Charpentier, E.; Mohamed, M.A.; Berry, A.; Aboubaker, M.H. Misdiagnosis of imported falciparum malaria from African areas due to an increased prevalence of pfhrp2/pfhrp3 gene deletion: the Djibouti case. *Emerg Microbes Infect* **2020**, *9*, 1984-1987, doi:10.1080/22221751.2020.1815590.
8. Schindler, T.; Deal, A.C.; Fink, M.; Guirou, E.; Moser, K.A.; Mwakasungula, S.M.; Mihayo, M.G.; Jongo, S.A.; Chaki, P.P.; Abdulla, S.; et al. A multiplex qPCR approach for detection of pfhrp2 and pfhrp3 gene deletions in multiple strain infections of Plasmodium falciparum. *Sci Rep* **2019**, *9*, 13107, doi:10.1038/s41598-019-49389-2.
9. Berzosa, P.; Gonzalez, V.; Taravillo, L.; Mayor, A.; Romay-Barja, M.; Garcia, L.; Ncogo, P.; Riloha, M.; Benito, A. First evidence of the deletion in the pfhrp2 and pfhrp3 genes in Plasmodium falciparum from Equatorial Guinea. *Malar J* **2020**, *19*, 99, doi:10.1186/s12936-020-03178-9.

10. Hosch, S.; Yoboue, C.A.; Donfack, O.T.; Guirou, E.A.; Dangy, J.P.; Mpina, M.; Nyakurungu, E.; Blochliger, K.; Guerra, C.A.; Phiri, W.P.; et al. Analysis of nucleic acids extracted from rapid diagnostic tests reveals a significant proportion of false positive test results associated with recent malaria treatment. *Malar J* **2022**, *21*, 23, doi:10.1186/s12936-022-04043-7.
11. Berhane, A.; Anderson, K.; Mihreteab, S.; Gresty, K.; Rogier, E.; Mohamed, S.; Hagos, F.; Embaye, G.; Chinorumba, A.; Zehaie, A.; et al. Major Threat to Malaria Control Programs by Plasmodium falciparum Lacking Histidine-Rich Protein 2, Eritrea. *Emerg Infect Dis* **2018**, *24*, 462-470, doi:10.3201/eid2403.171723.
12. Girma, S.; Cheaveau, J.; Mohon, A.N.; Marasinghe, D.; Legese, R.; Balasingam, N.; Abera, A.; Feleke, S.M.; Golassa, L.; Pillai, D.R. Prevalence and Epidemiological Characteristics of Asymptomatic Malaria Based on Ultrasensitive Diagnostics: A Cross-sectional Study. *Clin Infect Dis* **2019**, *69*, 1003-1010, doi:10.1093/cid/ciy1005.
13. Golassa, L.; Messele, A.; Amambua-Ngwa, A.; Swedberg, G. High prevalence and extended deletions in Plasmodium falciparum hrp2/3 genomic loci in Ethiopia. *PLoS One* **2020**, *15*, e0241807, doi:10.1371/journal.pone.0241807.
14. Feleke, S.M.; Reichert, E.N.; Mohammed, H.; Brhane, B.G.; Mekete, K.; Mamo, H.; Petros, B.; Solomon, H.; Abate, E.; Hennelly, C.; et al. Plasmodium falciparum is evolving to escape malaria rapid diagnostic tests in Ethiopia. *Nat Microbiol* **2021**, *6*, 1289-1299, doi:10.1038/s41564-021-00962-4.
15. Alemayehu, G.S.; Blackburn, K.; Lopez, K.; Cambel Dieng, C.; Lo, E.; Janies, D.; Golassa, L. Detection of high prevalence of Plasmodium falciparum histidine-rich protein 2/3 gene deletions in Assosa zone, Ethiopia: implication for malaria diagnosis. *Malar J* **2021**, *20*, 109, doi:10.1186/s12936-021-03629-x.
16. Rogier, E.; McCaffery, J.N.; Nace, D.; Svigel, S.S.; Assefa, A.; Hwang, J.; Kariuki, S.; Samuels, A.M.; Westercamp, N.; Ratsimbasoa, A.; et al. Plasmodium falciparum pfhrp2 and pfhrp3 Gene Deletions from Persons with Symptomatic Malaria Infection in Ethiopia, Kenya, Madagascar, and Rwanda. *Emerg Infect Dis* **2022**, *28*, 608-616, doi:10.3201/eid2803.211499.
17. Leonard, C.M.; Assefa, A.; McCaffery, J.N.; Herman, C.; Plucinski, M.; Sime, H.; Mohammed, H.; Kebede, A.; Solomon, H.; Haile, M.; et al. Investigation of Plasmodium falciparum pfhrp2 and pfhrp3 gene deletions and performance of a rapid diagnostic test for identifying asymptomatic malaria infection in northern Ethiopia, 2015. *Malar J* **2022**, *21*, 70, doi:10.1186/s12936-022-04097-7.
18. Nderu, D.; Kimani, F.; Thiong'o, K.; Akinyi, M.; Karanja, E.; Meyer, C.G.; Velavan, T.P. PfHRP2-PfHRP3 diversity among Kenyan isolates and comparative evaluation of PfHRP2/pLDH malaria RDT with microscopy and nested PCR methodologies. *Parasitol Int* **2018**, *67*, 793-799, doi:10.1016/j.parint.2018.08.007.
19. Sepulveda, N.; Phelan, J.; Diez-Benavente, E.; Campino, S.; Clark, T.G.; Hopkins, H.; Sutherland, C.; Drakeley, C.J.; Beshir, K.B. Global analysis of Plasmodium falciparum histidine-rich protein-2 (pfhrp2) and pfhrp3 gene deletions using whole-genome sequencing data and meta-analysis. *Infect Genet Evol* **2018**, *62*, 211-219, doi:10.1016/j.meegid.2018.04.039.
20. Nderu, D.; Kimani, F.; Thiong'o, K.; Karanja, E.; Akinyi, M.; Too, E.; Chege, W.; Nambati, E.; Meyer, C.G.; Velavan, T.P. Plasmodium falciparum histidine-rich protein (PfHRP2 and 3) diversity in Western and Coastal Kenya. *Sci Rep* **2019**, *9*, 1709, doi:10.1038/s41598-018-38175-1.
21. Grignard, L.; Nolder, D.; Sepulveda, N.; Berhane, A.; Mihreteab, S.; Kaaya, R.; Phelan, J.; Moser, K.; van Schalkwyk, D.A.; Campino, S.; et al. A novel multiplex qPCR assay for detection of Plasmodium falciparum with histidine-rich protein 2 and 3 (pfhrp2 and pfhrp3) deletions in polyclonal infections. *EBioMedicine* **2020**, *55*, 102757, doi:10.1016/j.ebiom.2020.102757.
22. Galatas, B.; Mayor, A.; Gupta, H.; Balanza, N.; Jang, I.K.; Nhamussua, L.; Simone, W.; Cistero, P.; Chidimatembue, A.; Munguambe, H.; et al. Field performance of ultrasensitive and conventional malaria rapid diagnostic tests in southern Mozambique. *Malar J* **2020**, *19*, 451, doi:10.1186/s12936-020-03526-9.
23. Funwei, R.; Nderu, D.; Nguetse, C.N.; Thomas, B.N.; Falade, C.O.; Velavan, T.P.; Ojurongbe, O. Molecular surveillance of pfhrp2 and pfhrp3 genes deletion in Plasmodium falciparum isolates and the implications for rapid diagnostic tests in Nigeria. *Acta Trop* **2019**, *196*, 121-125, doi:10.1016/j.actatropica.2019.05.016.
24. Prosser, C.; Gresty, K.; Ellis, J.; Meyer, W.; Anderson, K.; Lee, R.; Cheng, Q. Plasmodium falciparum Histidine-Rich Protein 2 and 3 Gene Deletions in Strains from Nigeria, Sudan, and South Sudan. *Emerg Infect Dis* **2021**, *27*, 471-479, doi:10.3201/eid2702.191410.
25. Thomson, R.; Beshir, K.B.; Cunningham, J.; Baiden, F.; Bharmal, J.; Bruxvoort, K.J.; Maiteki-Sebuguzi, C.; Owusu-Agyei, S.; Staedke, S.G.; Hopkins, H. pfhrp2 and pfhrp3 Gene Deletions That Affect Malaria Rapid Diagnostic Tests for Plasmodium falciparum: Analysis of Archived Blood Samples From 3 African Countries. *J Infect Dis* **2019**, *220*, 1444-1452, doi:10.1093/infdis/jiz335.
26. Bakari, C.; Jones, S.; Subramaniam, G.; Mandara, C.I.; Chiduo, M.G.; Rumisha, S.; Chacky, F.; Molteni, F.; Mandike, R.; Mkude, S.; et al. Community-based surveys for Plasmodium falciparum pfhrp2 and pfhrp3 gene deletions in selected regions of mainland Tanzania. *Malar J* **2020**, *19*, 391, doi:10.1186/s12936-020-03459-3.
27. Kaaya, R.D.; Kavishe, R.A.; Tenu, F.F.; Matowo, J.J.; Mosha, F.W.; Drakeley, C.; Sutherland, C.J.; Beshir, K.B. Deletions of the Plasmodium falciparum histidine-rich protein 2/3 genes are common in field isolates from north-eastern Tanzania. *Sci Rep* **2022**, *12*, 5802, doi:10.1038/s41598-022-09878-3.

28. Agaba, B.B.; Anderson, K.; Gresty, K.; Prosser, C.; Smith, D.; Nankabirwa, J.I.; Nsobya, S.; Yeka, A.; Opigo, J.; Gonahasa, S.; et al. Molecular surveillance reveals the presence of pfhrp2 and pfhrp3 gene deletions in Plasmodium falciparum parasite populations in Uganda, 2017-2019. *Malar J* **2020**, *19*, 300, doi:10.1186/s12936-020-03362-x.
29. Nsobya, S.L.; Walakira, A.; Namirembe, E.; Kiggundu, M.; Nankabirwa, J.I.; Ruhamyankaka, E.; Arinaitwe, E.; Conrad, M.D.; Kamya, M.R.; Dorsey, G.; et al. Deletions of pfhrp2 and pfhrp3 genes were uncommon in rapid diagnostic test-negative Plasmodium falciparum isolates from Uganda. *Malar J* **2021**, *20*, 4, doi:10.1186/s12936-020-03547-4.
30. Kobayashi, T.; Sikalima, J.; Parr, J.B.; Chaponda, M.; Stevenson, J.C.; Thuma, P.E.; Mulenga, M.; Meshnick, S.R.; Moss, W.J.; For The, S.; et al. The Search for Plasmodium falciparum histidine-rich protein 2/3 Deletions in Zambia and Implications for Plasmodium falciparum histidine-rich protein 2-Based Rapid Diagnostic Tests. *Am J Trop Med Hyg* **2019**, *100*, 842-845, doi:10.4269/ajtmh.18-0859.
31. Gibbons, J.; Qin, J.; Malla, P.; Wang, Z.; Brashear, A.; Wang, C.; Miao, J.; Adams, J.H.; Kim, K.; Jiang, R.; et al. Lineage-Specific Expansion of Plasmodium falciparum Parasites With pfhrp2 Deletion in the Greater Mekong Subregion. *J Infect Dis* **2020**, *222*, 1561-1569, doi:10.1093/infdis/jiaa250.
32. Pati, P.; Dhangadamajhi, G.; Bal, M.; Ranjit, M. High proportions of pfhrp2 gene deletion and performance of HRP2-based rapid diagnostic test in Plasmodium falciparum field isolates of Odisha. *Malar J* **2018**, *17*, 394, doi:10.1186/s12936-018-2502-3.
33. Kumari, S.; Ahmed, M.Z.; Sharma, S.; Pande, V.; Anvikar, A.R. Prevalence of Pfhrp2/3 gene deletions among false negative rapid antigen test results in central India. *J Vector Borne Dis* **2021**, *58*, 273-280, doi:10.4103/0972-9062.328815.
34. Kumari, M.S.; Sharma, S.; Bhardwaj, N.; Kumar, S.; Ahmed, M.Z.; Pande, V.; Anvikar, A.R. Pfhrp2/3 gene deletion and genetic variation in PfHRP2-based RDTs with P. falciparum positive samples from India and its implication on malaria control. *Infect Genet Evol* **2022**, *99*, 105232, doi:10.1016/j.meegid.2022.105232.
35. Goes, L.; Chamma-Siqueira, N.; Peres, J.M.; Nascimento, J.M.; Valle, S.; Arcanjo, A.R.; Lacerda, M.; Blume, L.; Povoa, M.; Viana, G. Evaluation of Histidine-Rich Proteins 2 and 3 Gene Deletions in Plasmodium falciparum in Endemic Areas of the Brazilian Amazon. *Int J Environ Res Public Health* **2020**, *18*, doi:10.3390/ijerph18010123.
36. Fontecha, G.; Mejia, R.E.; Banegas, E.; Ade, M.P.; Mendoza, L.; Ortiz, B.; Sabillon, I.; Alvarado, G.; Matamoros, G.; Pinto, A. Deletions of pfhrp2 and pfhrp3 genes of Plasmodium falciparum from Honduras, Guatemala and Nicaragua. *Malar J* **2018**, *17*, 320, doi:10.1186/s12936-018-2470-7.
37. Nolder, D.; Stewart, L.; Tucker, J.; Ibrahim, A.; Gray, A.; Corrah, T.; Gallagher, C.; John, L.; O'Brien, E.; Aggarwal, D.; et al. Failure of rapid diagnostic tests in Plasmodium falciparum malaria cases among travelers to the UK and Ireland: Identification and characterisation of the parasites. *Int J Infect Dis* **2021**, *108*, 137-144, doi:10.1016/j.ijid.2021.05.008.
